# A cross-sectional study of parental awareness of and reasons for lack of health insurance among minority children, and the impact on health, access to care, and unmet needs

**DOI:** 10.1186/s12939-016-0331-y

**Published:** 2016-03-22

**Authors:** Glenn Flores, Hua Lin, Candy Walker, Michael Lee, Alberto Portillo, Monica Henry, Marco Fierro, Kenneth Massey

**Affiliations:** Medica Research Institute, MR-CW105, P.O. Box 9310, Minneapolis, MN 55440-9310 USA; Department of Clinical Sciences, UT Southwestern Medical Center, 5323 Harry Hines Blvd., Dallas, TX USA; Texas Scottish Rite Hospital for Children, 2222 Welborn St, Dallas, TX 75219 USA; Department of Pediatrics, UT Southwestern Medical Center, 5323 Harry Hines Blvd., Dallas, TX 75390-9063 USA; Children’s Health System of Texas, Dallas, TX 75235 USA

**Keywords:** Child, Adolescent, Medically uninsured, Public health, Hispanic Americans, African-Americans

## Abstract

**Background:**

Minority children have the highest US uninsurance rates; Latino and African-American children account for 53 % of uninsured American children, despite comprising only 48 % of the total US child population. The study aim was to examine parental awareness of and the reasons for lacking health insurance in Medicaid/CHIP-eligible minority children, and the impact of the children’s uninsurance on health, access to care, unmet needs, and family financial burden.

**Methods:**

For this cross-sectional study, a consecutive series of uninsured, Medicaid/CHIP-eligible Latino and African-American children was recruited at 97 urban Texas community sites, including supermarkets, health fairs, and schools. Measures/outcomes were assessed using validated instruments, and included sociodemographic characteristics, uninsurance duration, reasons for the child being uninsured, health status, special healthcare needs, access to medical and dental care, unmet needs, use of health services, quality of care, satisfaction with care, out-of-pocket costs of care, and financial burden.

**Results:**

The mean time uninsured for the 267 participants was 14 months; 5 % had never been insured. The most common reason for insurance loss was expired and never reapplied (30 %), and for never being insured, high insurance costs. Only 49 % of parents were aware that their uninsured child was Medicaid/CHIP eligible. Thirty-eight percent of children had suboptimal health, and 2/3 had special healthcare needs, but 64 % have no primary-care provider; 83 % of parents worry about their child’s health more than others. Unmet healthcare needs include: healthcare, 73 %; mental healthcare, 70 %; mobility aids/devices, 67 %; dental, 61 %; specialty care, 57 %; and vision, 46 %. Due to the child’s health, 35 % of parents had financial problems, 23 % cut work hours, and 10 % ceased work. Higher proportions of Latinos lack primary-care providers, and higher proportions of African-Americans experience family financial burden.

**Conclusions:**

Half of parents of uninsured minority children are unaware that their children are Medicaid/CHIP-eligible. These uninsured children have suboptimal health, impaired access to care, and major unmet needs. The child’s health causes considerable family financial burden, and one in 10 parents ceased work. The study findings indicate urgent needs for better parental education about Medicaid/CHIP, and for improved Medicaid/CHIP outreach and enrollment.

## Background

Although the number and proportion of US children without health insurance modestly declined between 2008-2012 [1], 6 % of US children (4.8 million) are uninsured [2], and 68 % (3.3 million) are eligible for but not enrolled in Medicaid or the Children’s Health Insurance Program (CHIP) [3]. Among uninsured US children, racial/ethnic disparities exist. In contrast to only 4.9 % of white children being uninsured, 9.6 % of Latino and 5.1 % of African-American children are uninsured [2]. Indeed, Latino and African-American children account for 53 % of uninsured American children [4], despite comprising only 48 % of the total population of US children [5].

Although 2.4 million Latino and African-American children are uninsured [4], not enough is known about the health and healthcare impact on these children of uninsurance, particularly from studies analyzing primary (rather than administrative/secondary) data. For the 1.6 million uninsured Latino and African-American children eligible for but not enrolled in Medicaid/CHIP, it is unknown what proportion of parents are aware of their children’s Medicaid/CHIP eligibility, and the reasons why these children remain uninsured. The study aims, therefore, were to identify the health and healthcare impact, parental awareness of uninsured children’s Medicaid/CHIP eligibility, and reasons why children lost or never had health insurance, among uninsured minority children eligible for but not enrolled in Medicaid/CHIP. Specifically, the objective was to use uniquely extensive primary data on a sample of uninsured minority children eligible for but not enrolled in Medicaid/CHIP to assess: parental sociodemographic features and health-insurance characteristics, and children’s sociodemographic features, health insurance, reasons for loss of health insurance and never having health insurance, health status, special healthcare needs (SHCNs), access to healthcare, unmet healthcare needs, use of health services, out-of-pocket healthcare costs, quality of care, quality of life, parental satisfaction with care, financial burden of children’s healthcare, and missed school and work due to the child’s health.

## Methods

### Study design and participants

In this cross-sectional study, a consecutive series of uninsured Latino and African-American children 0-18 years old residing in Dallas County who were eligible for but not enrolled in Medicaid/CHIP was recruited from June 2011 to January 2014 at 97 community sites in regions with the highest proportion of uninsured and low-income residents. To avoid distortion due to clustering, only one uninsured child per family was enrolled. To ensure participants were representative of families with uninsured children in these regions, recruitment occurred in a variety of community settings, including supermarkets, department stores, dollar stores, Goodwill stores, restaurants, public libraries, community centers, food banks, health fairs, Boys and Girls clubs, YMCAs, churches, schools, community outpatient clinics, day-care establishments, Laundromats, apartment complexes, housing projects, homeless shelters, and WIC centers. Participant eligibility criteria included: 1) children 0-18 years old currently lacking health insurance; 2) parental self-identification of the child as Latino/Hispanic, African-American/black, or both; and 3) the child is eligible for Medicaid or CHIP. Eligibility for Medicaid or CHIP initially was determined in the field by research staff using family income, assets, and other information required to determine eligibility by the State of Texas, and then officially confirmed through electronic verification by the Texas Health and Human Services Commission, which administers both Medicaid and CHIP for the state. Subjects subsequently participated in a randomized trial, called Kids’ HELP (**Kids**' **H**ealth Insurance by **E**ducating **L**ots of **P**arents), of the effects of parent mentors on insuring uninsured minority children, full details of which are available elsewhere [6].

Dallas County, TX, is an ideal setting for studying uninsured minority children, because: 1) Texas has the highest proportion and number of uninsured of any state in America, at 19 % and five million, respectively; 2) Texas has the highest number of uninsured children (783,938); and 3) 15.9 % of children in Dallas County are uninsured, compared with 9 % in Texas and 6 % in the US [7–9].

All participants provided written consent and/or assent (when indicated). This study was approved by the University of Texas Southwestern Institutional Review Board.

### Surveys

Primary caregivers (as identified by families) completed eligibility screening surveys (using Texas Medicaid/CHIP eligibility criteria [10]) and two questionnaires (in English or Spanish, according to parental preference) orally administered by trained bilingual research assistants, to overcome language and literacy barriers. The conceptual framework for the questionnaire domains and questions was a slightly modified version of the World Health Organization’s social-determinants-of-health framework [11]: specifically, survey items focused on social determinants of health, defined as the conditions in which people are born, grow, live, work, and age, and which are considered primarily responsible for health inequities. The first questionnaire consisted of 82 questions addressing parental and children’s characteristics; whenever possible, validated questions from longstanding national and state surveys and published articles were used [12–25]. Fourteen questions addressed parental sociodemographics and health insurance, and 68 examined children’s sociodemographics, health insurance, reasons for loss of health insurance and never having health insurance, health status, SHCNs, access to healthcare, unmet healthcare needs, use of health services, out-of-pocket healthcare costs, quality of care, parental satisfaction with care, financial burden of children’s healthcare, and missed school and work due to the child’s health. The Maternal and Child Health Bureau’s standard definition for SHCNs was used (those who have a chronic physical, developmental, behavioral, or emotional condition and who also require health and related services of a type or amount beyond that required by children generally) [26]. Children’s quality of life was assessed using the Pediatric Quality of Life Inventory Version 4.0 Generic Core Scales (PedsQL) [27]. Responses to open-ended questions were categorized by theme, and frequencies of response themes tallied. Complete details on definitions, outcomes, and survey instruments are available elsewhere [6].

Parental awareness of the uninsured child’s Medicaid/CHIP eligibility was assessed with the yes/no question, “Your child is eligible for Medicaid or CHIP. Did you know that?” For affirmative responses, parents also were asked the open-ended question, “Why have you chosen not to enroll your child in Medicaid or CHIP?” Although implementation of the Affordable Care Act (ACA) occurred in only the final month of the 32 months of data collection, parents were asked whether the ACA affected their awareness and knowledge of children’s Medicaid/CHIP, and none reported any ACA impact.

### Analyses

SAS 9.3 was used for all analyses, with a two-tailed *P* < .05 considered statistically significant. To summarize parental and child characteristics, percentages were used for categorical data and means for continuous data. Bivariate analyses identified racial/ethnic differences and factors associated with parental awareness of Medicaid/CHIP eligibility. The non-parametric Wilcoxon test was used to evaluate continuous variables, and the Pearson Chi-square test for categorical outcomes. For the hierarchical multivariable logistic regression analysis of factors associated with parental unawareness of children’s Medicaid/CHIP eligibility, SAS Proc Logistic was used, with stepwise selection and an initial alpha-to-enter of 0.15 [28].

## Results

### Sample characteristics

**Fig. 1:**
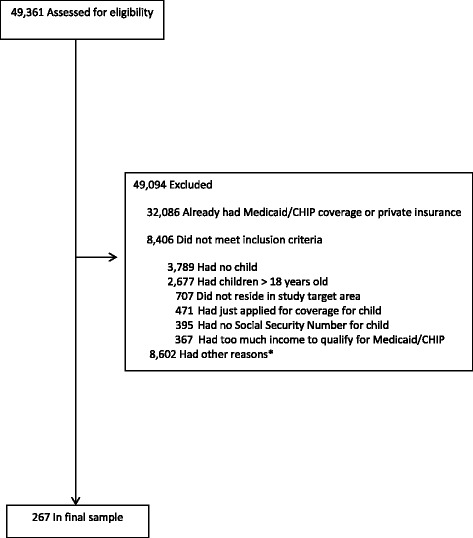
Participant accrual and reasons for exclusion of ineligible study candidates. *Including not interested in participating in study, took information without further follow-up, legal-custody issues, and grandparent did not know whether or not the child was insured

We screened 49,361 caregivers; 49,094 candidates were excluded, due to failure to meet inclusion criteria, children already having Medicaid/CHIP or private insurance, and other reasons, yielding the final sample of 267 children (Fig. 1). The mean child age was seven years old, half were female, about 2/3 were Latino, 1/3 were African-American, and most were US-born (Table 1). Primary caregivers (hereafter referred to as parents) were predominantly female; 1/3 had limited English proficiency (LEP), the plurality were married and living with the spouse, >40 % were not high-school graduates, and > ½ were unemployed. About ¼ of parents had health insurance, primarily Medicaid. The mean annual family income was $21,857. There were means of two children and two adults in the households.

**Table 1:** Selected characteristics of uninsured minority children and their parents (*N* = 267)

Characteristic	Mean or %
Age of child (years), mean (range)	7.3 (1, 18)
Gender of child	
Male	50 %
Female	50 %
Race/ethnicity of child	
Latino	65 %
African-American	35 %
Child born in US	95 %
Gender of primary caregiver	
Female	96 %
Male	4 %
Primary caregiver has limited English proficiency	32 %
Marital status of primary caregiver	
Married, living with spouse	39 %
Single	30 %
Married, separated from spouse	13 %
Common-law marriage	7 %
Divorced	6 %
Living with partner	4 %
Widowed	1 %
Primary caregiver not high-school graduate	43 %
Primary caregiver unemployed	53 %
Primary caregiver has health insurance	26 %
Type of insurance coverage for insured caregiver	
Public	52 %
Private	45 %
Other	3 %
Mean combined annual family income (range)	$21,857 ($1,440, $64,000)
Mean number of children in household (range)	2.3 (1, 13)
Mean number of adults in household (range)	2.1 (1, 6)
Primary caregiver aware that child is eligible for Medicaid or CHIP	49 %
Child ever had health insurance before	95 %
Insurance that uninsured child had in past	
Medicaid	72 %
CHIP	14 %
Private	13 %
Instituto Mexicano del Seguro Social^a^	1 %
Mean months without insurance (range)	14.2 (1-144)
Median months without insurance (inner 95th percentile range)	6 (1, 84)
Reason child lost insurance^b^	
Insurance expired and never reapplied	30 %
Was told making too much income/did not qualify	13 %
Changed job	13 %
Change of address	11 %
Applied but never got reply or information	9 %
Missing paperwork	8 %
Does not know	6 %
Father was supposed to cover child	4 %
Too expensive	2 %
Did not provide reason	2 %
Child was determined to be disabled	1 %
Sometimes paid premium late and was dropped	1 %
Language barrier	1 %
Insurance stopped when child was adopted	1 %
Child not US citizen	1 %
Told child aged out of health-insurance coverage	1 %
Not legal guardian	1 %

^a^Public health insurance in Mexico

^b^Cited by parents of uninsured minority children (*N* = 257) who were eligible for but not enrolled in Medicaid or CHIP. Does not include 12 children who have never been insured during their lifetime. Proportions sum to > 100 % because parents could choose more than one reason

Less than ½ of parents were aware their uninsured child was Medicaid/CHIP eligible (Table 1). Ninety-five percent of children ever had insurance before, most commonly Medicaid (almost ¾ of children), followed by CHIP, private insurance, and Mexico’s public health insurance. The mean months without health insurance was 14, ranging from 1 month to 12 years, with a median of six months uninsured.

### Reasons for insurance loss/never having insurance

Insurance coverage most commonly was lost due to insurance expiration without parents ever reapplying, in 1/3 of cases (Table 1). The next most common reasons were the family had too much income/did not qualify, changing jobs, change of address, and applied but never received a subsequent reply. The remaining 12 reasons were cited by <9 % of parents.

For the 5 % of children never insured during their lifetime, the most common reasons for the child never being covered included the high cost of insurance (1/4), and language barriers, moving to Texas, and hassles, each at 15 %. Other reasons included originally not a documented citizen, being told the child is ineligible due to parental employment, applied but never heard back, and no reason, each at 8 %. Thus, in contrast to children with previous insurance coverage, unique reasons for those never covered include a move to Texas, hassles, and being told parental employment makes children ineligible.

### Factors associated with parental unawareness

Several factors were associated with parental unawareness of uninsured children’s Medicaid/CHIP eligibility (Table 2). The mean duration of uninsurance was longer for unaware vs. aware parents (17 vs. 12 months), and the mean family income was higher by almost $4,000. Latino parents (57 %) were more likely to be unaware than African-American parents (40 %). Racial/ethnic differences in awareness, however, disappeared after adjustment for uninsurance duration and income (Table 3).

**Table 2:** Bivariate analysis of factors associated with parental unawareness of their uninsured child’s eligibility for Medicaid or CHIP

Characteristic	Unaware of child’s medicaid/CHIP eligibility^a^	Aware of child’s medicaid/CHIP eligibility	*P*
Mean time uninsured, months	16.8	11.8	.01
Mean annual combined family			
income	$23,777	$19,871	.01
Race/ethnicity			.04
Latino	57.1 %	42.9 %	
African-American	40.4 %	59.6 %	

^a^No other factors were significantly associated with unawareness of child’s insurance eligibility

**Table 3:** Multivariable hierarchical logistic regression analysis of factors associated with parental unawareness of their uninsured child’s eligibility for Medicaid or CHIP^a^

	Odds Ratio (95 % CI) of Parental Unawareness of Uninsured Child’s Eligibility for Medicaid/CHIP		
Characteristic	Model 1	Model 2	Model 3
Latino race/ethnicity^b^	**2.0 (1.2, 3.3)**	1.7 (0.99, 2.9)	1.6 (0.9, 2.8)
Mean annual combined family income	—	1.0 (0.99, 1.0)	1.0 (0.99, 1.0)
Mean months uninsured	—	—	0.98 (0.98, 1.0)

^a^All sociodemographic characteristics were eligible for model entry, but those listed are the only factors in the final model. Bold denotes a statistically significant odds ratio.

^b^Referent: African-American/black race/ethnicity

### Health status and SHCNs

Over one-third of children had not excellent/very good health status (Table 4). More than four in five parents reported worrying about their child’s health more than other people, and ¾ expressed worry/concern about the child’s health. About 2/3 of children have SHCNs, consisting of 17 conditions, including particularly high rates (≥12 %) of eczema, respiratory allergies, asthma, behavior problems, and bone/joint conditions. In addition to these five conditions, noteworthy among the remaining 12 conditions include a 5-7 % prevalence of attention-deficit-hyperactivity disorder (ADHD), speech problems, depression, and anxiety; a 1-2 % prevalence of hearing problems, autism, and brain injury; and > ¼ with other health problems.

**Table 4:** Health status, special healthcare needs, access to healthcare, unmet healthcare needs,^a^ use of health services, parental out-of-pocket costs of health services, quality of pediatric care, parental satisfaction with pediatric care, parental-reported financial burden of child’s healthcare, and missed school and work days due to child’s health among uninsured minority children eligible for but not insured by Medicaid or CHIP (*N* = 267)

Measure	Proportion, Mean, or Mean Cost
Health status	
Health status not excellent/very good	38 %
Worry about child’s health more than other people	83 %
Have emotional worry or concern about child’s physical health	75 %
Special healthcare need	
Any special healthcare need	66 %
Eczema	28 %
Respiratory allergy	18 %
Asthma	15 %
Behavior problem	13 %
Bone or joint problem	12 %
More than three ear infections	7 %
Allergy	7 %
ADHD	7 %
Speech problem	7 %
Depression	6 %
Headache	6 %
Anxiety	5 %
Hearing problem	2 %
Autism	1 %
Brain injury	1 %
Diabetes	0.4 %
Any other health problem	27 %
Access to healthcare	
Child has no PCP	64 %
Child has no usual source of preventive care	40 %
Sources of preventive care and sick care different	55 %
Never/sometimes gets immediate care from PCP	10 %
Usual source of care has no night or weekend office hours	88 %
Does not have access to 24-hour telephone coverage for sick care	88 %
No access to same-day sick-care visit without appointment	67 %
Child has no usual source of sick care	18 %
Difficult getting appointment for sick care	12 %
Never/sometimes has access to interpreter^b^	11 %
Has problems getting care from specialists	8 %
Has problem getting special services^c^	8 %
Never/sometimes can obtain after-hours telephone help or advice	6 %
Unmet healthcare needs	
Delayed or didn’t get needed healthcare	73 %
Didn’t receive all needed dental care	61 %
Didn’t receive all needed preventive care	51 %
Didn’t receive all needed hearing aids or hearing care	100 %
Didn’t receive all needed physical, occupational, or speech therapy	79 %
Didn’t receive all needed mental healthcare	70 %
Didn’t receive all needed mobility aids or devices^d^	67 %
Didn’t receive all needed specialty care	57 %
Problems obtaining needed specialty referral	53 %
Didn’t receive all needed home healthcare	50 %
Didn’t receive all needed vision care	46 %
Didn’t receive all needed medical supplies or equipment^e^	23 %
Didn’t receive all needed acute care	19 %
Didn’t receive all needed prescription medications	18 %
Use and out-of-pocket cost of health services	
Mean number of any doctor visits in past year	3.3
Mean out-of-pocket cost per doctor visit^f^	$141.7
Mean number of preventive care visits in past year	1.0
Mean out-of-pocket cost per preventive-care visit^g^	$46.7
Mean number of sick visits in past year	1.8
Mean out-of-pocket cost per sick visit^h^	$195.1
Mean number of ED visits in past year	0.9
Mean out-of-pocket cost per ED visit^i^	$421.0
Mean number of hospital stays in past year	0.1
Mean out-of-pocket cost per hospital stay^j^	$593.1
Parental-reported financial burden of child’s healthcare	
Need additional income to cover child’s medical expenses	45 %
Child’s health caused financial problems for family	35 %
Family cut down on work hours to obtain healthcare for child	23 %
Stopped working because of child’s health	10 %
Missed school and work days due to child’s health	
Mean missed school days due to child’s illness	3.4
Mean missed work days due to child’s illness	1.7
Mean wage loss due to missed work days for child’s illness^k^	$342.9
Other mean costs related to taking care of sick child^k^	$155.5
Quality of care^l^	
Mean rating of child’s well-child visits	8.3
Mean rating of quality of child’s primary-care provider	8.9
Mean rating of quality of child’s acute care	8.6
Mean rating of quality of child’s specialty provider	8.3
Parental satisfaction with child’s pediatric care	
Doctor never/sometimes asks how you are feeling as parent	40 %
Doctor never/sometimes understands how your prefer to raise child	27 %
Doctor never/sometimes take time to understand child’s specific needs	21 %
Doctor never/sometimes respect you are expert on your child	16 %
Did not ask all questions I wanted to ask doctor	15 %
Doctor didn’t spend enough time with child	14 %
Would not recommend child’s healthcare provider to friends	24 %
Quality of life (mean PedsQL score)	
Total	91
Physical functioning	93
Social functioning	92
Emotional functioning	86
School functioning	85

^a^The denominator for each unmet healthcare need consists of only those parents responding that their child needed the particular category of care or service

^b^Only among parents who had limited English proficiency

^c^Includes physical therapy, medical equipment (such as wheelchairs), special-education services, or counseling

^d^Includes canes, crutches, wheelchairs, or scooters

^e^Such as G-tubes and nebulizer machines

^f^Among children who made any doctor visit

^g^Among children who made any preventive visit

^h^Among children who made any sick visit

^i^Among children who made any ED visit

^j^Among children who were hospitalized

^k^Among parents who missed any work due child’s illness

^l^On scale of 0-10, where 0 = worst and 10 = best

### Access and unmet needs

Substantial access barriers to healthcare and unmet needs were identified (Table 4). Approximately 2/3 of children have no primary-care provider (PCP), 40 % have no usual preventive-care source, >½ have different sources of preventive and sick care, and one in 10 never/sometimes gets immediate care from the PCP. About nine in 10 have a usual source of care that has no night or weekend hours and have no access to 24-h phone coverage for sick care, 2/3 have no access to same-day sick-care visits without appointments, one in five has no usual sick-care source, one in eight has difficulty obtaining sick-care appointments, and 11 % of LEP parents never/sometimes have access to medical interpreters. One in 12 children needing specialty care experienced problems obtaining specialty care, 8 % of those needing special services encountered problems obtaining those services, and 6 % of parents are never/sometimes able to obtain after-hours telephone help/advice.

Almost ¾ of parents delayed/didn’t get needed healthcare for their children (Table 4). Almost two-thirds of children had unmet dental-care needs, and > ½ had unmet preventive-care needs. All children with auditory deficits had unmet needs for hearing aids/hearing care. Among children with specific needs, >2/3 had unmet needs for physical/occupational/speech therapy; mental healthcare; and mobility aids/devices. Almost 60 % of children had unmet specialty-care needs, and > ½ had problems obtaining specialty referrals. Approximately half of children had unmet needs for home healthcare and vision care. About ¼ of children needing medical supplies/equipment did not receive them, and one in five children had unmet needs for acute care and prescription medications.

### Use of services, financial burden, and missed school and work

Children averaged three physician visits, one preventive visit, two sick visits, one ED visit, and 0.1 hospital stays annually (Table 4). Mean out-of-pocket costs ranged from $47/preventive visit to $593/hospitalization.

Almost ½ of parents reported needing additional income to cover children’s medical expenses, and >1/3 that the child’s health caused family financial problems (Table 4). Approximately ¼ of parents reported the family cut work hours to obtain healthcare for children, and one in 10 stopped working because of their child’s health.

Children missed a mean of three school days, and parents a mean of two work days annually due to children’s illnesses (Table 4). The mean wage loss was $343/missed work day, and families also incurred mean additional costs of $156 in the past year due to children’s illnesses.

### Quality of care, satisfaction with care, and quality of life

Mean quality-of-care ratings ranged from 8-9 (Table 4). Forty percent of parents reported children’s doctor never/sometimes asks how they are feeling as parents, and > ¼ that doctors never/sometimes understand how they prefer to raise their child (Table 4). Between 14-21 % of parents reported the doctor never/sometimes understands children’s specific needs or respects the parent as the expert on the child, the doctor didn’t spend enough time with the child, and the parent did not ask doctors all questions he/she wanted. Almost ¼ of parents would not recommend their child’s healthcare provider to friends. The overall mean PedsQL score was 91 (Table 4).

### Racial/ethnic differences

A higher proportion of Latinos report worrying about their child’s health more than others, at almost 90 % vs. 3/4 of African-Americans (Table 5). Almost ¾ of Latino children have no PCP, vs. about ½ of African-American children, and Latino children are more likely to have no usual preventive-care source, and different sources of preventive and acute care. Almost all Latino children have usual sources of care without night or weekend hours and lacking 24-h phone coverage for sick care, vs. approximately ¾ of African-American children for both. Mean out-of-pocket costs per doctor visit and per sick visit and the number of yearly sick visits also were greater for Latino children.

**Table 5:** Racial/ethnic differences in health status, special healthcare needs, access to healthcare, unmet healthcare needs, use of health services, parental out-of-pocket costs of health services, parental satisfaction with pediatric care, and parental-reported financial burden of child’s healthcare among uninsured minority children eligible for but not insured by Medicaid or CHIP (*N* = 267)^a^

Measure	Latinos (*N* = 174)	African-Americans (*N* = 93)	*P*
Health status			
Worry about child’s health more than other people	89 %	73 %	<.01
Special healthcare need			
Asthma	10 %	25 %	<.01
ADHD	4 %	12 %	.02
Access to healthcare			
Has no PCP	70 %	52 %	<.01
No usual source of preventive care	51 %	23 %	<.01
Source of sick care and preventive care different	60 %	45 %	.02
Usual source of care has no night or weekend office hours	93 %	79 %	<.01
Doesn’t have 24-hour phone coverage for sick care	95 %	75 %	<.01
No access to same-day sick-care visit without appointment	27 %	43 %	.01
Never/sometimes receive help or advice over phone	3 %	11 %	.01
Unmet healthcare needs			
Didn’t receive all needed acute care	5 %	18 %	.01
Didn’t receive all needed prescription medications	7 %	15 %	.03
Use and out-of-pocket costs of health services			
Mean out-of-pocket cost per doctor visit^b^	$153.2	$119.5	<.01
Mean out-of-pocket cost per preventive-care visit^c^	$50.0	$40.4	.03
Mean out-of-pocket cost per sick visit^d^	210.3	160.5	.01
Mean number of sick visits in past year	1.8	1.7	.04
Mean number of ED visits in past year	0.5	1.5	.03
Parental satisfaction with care			
Doctor never/sometimes understands how I prefer to raise my child	21 %	37 %	.01
Parental-reported financial burden			
Need additional income to cover child’s medical expenses	39 %	55 %	.02

^a^There were no significant racial/ethnic differences for any measure not listed in the table

^b^Among children who made any doctor visit

^c^Among children who made any preventive-care visit

^d^Among children who made any sick visit

African-American children are more likely than Latino children to have asthma and ADHD, no access to same-day sick care without appointments, to never/sometimes receive phone help/advice, and to make ED visits. African-American parents more often reported their child’s doctor never/sometimes understands how parents prefer to raise children, and > ½ need additional income to cover children’s medical expenses, vs. <40 % of Latino parents.

## Discussion

Over half of parents are unaware that their uninsured minority children are Medicaid/CHIP eligible. These new quantitative results complement prior qualitative work documenting that lack of Medicaid/CHIP awareness is a major reason why parents have not insured uninsured minority children [29, 30]. These qualitative studies shed light on the reasons for parental unawareness of uninsured children’s Medicaid/CHIP eligibility, including parents lacking basic knowledge about Medicaid/CHIP, their eligibility rules, and the application process; language and immigration issues; income verification obstacles; misinformation from insurance representatives; system impediments; and family mobility. The current study findings indicate lack of Medicaid/CHIP awareness remains a substantial barrier for the 3.3 million uninsured US children eligible for but not enrolled in Medicaid/CHIP, and suggest that enhancing awareness and outreach will be crucial to insuring more uninsured children. The data indicate that special efforts should be made to target populations at highest risk of parental unawareness of children’s Medicaid/CHIP eligibility, including those uninsured the longest, those at the higher end of income eligibility, and Latinos.

The results reveal that broad system issues also impede parents from insuring Medicaid/CHIP-eligible children. One in seven children lost insurance because Medicaid/CHIP representatives incorrectly told parents that their children did not qualify for these programs or parental income was excessive, although all children were Medicaid/CHIP eligible. Insurance representatives also incorrectly told some families that children had “aged out” of coverage. Remedying these serious system barriers to insuring uninsured children may require better training of insurance representatives and careful monitoring and reporting of such system glitches. In addition, 9 % of parents reported they had applied for Medicaid/CHIP for their children, but never received a reply, suggesting substantial room for system improvement in application tracking and responses.

Ninety-five percent of uninsured children previously had insurance, underscoring Medicaid/CHIP retention will continue to be especially important in eliminating childhood uninsurance. About ¾ of children previously had Medicaid coverage, indicating Medicaid loss is a considerable challenge, and perhaps reflecting Texas (at the time of this study) was one of two states still requiring Medicaid renewal every six months, and one of 27 states not providing 12-month continuous Medicaid eligibility (i.e., regardless of family-income fluctuations) [31]. Parents reported a panoply of reasons for children losing coverage, most commonly not reapplying after insurance expiration and not knowing why coverage was lost, suggesting better parental education is needed. It is concerning that system problems accounted for ¼ of children losing coverage. Although all children were Medicaid/CHIP eligible, 13 % incorrectly were told they were ineligible, and 9 % applied with no response from the Medicaid/CHIP program. Parents changing jobs and family moves comprised ¼ of the reasons why children lost insurance; such challenging impediments may require tailored interventions known to be effective in insuring uninsured minority children, such as community health workers [32] and parent mentors [33].

Both the duration without coverage and proportion of children never insured are troubling. Multiple studies document uninsured children are significantly more likely than insured children to have suboptimal health, no regular physician, delayed immunizations, unmet needs, impaired specialty access [34] and higher odds of ED visits [35], avoidable hospitalizations [36], injury hospitalizations [37], adverse newborn outcomes [38], and death in hospitals, ICUs, and after trauma [39, 40]. Our study population averaged 14 months without insurance, with some uninsured up to 12 years, placing many at high risk for these deleterious consequences, with the one in 20 children never insured during their lifetime at especially high risk. Parental reasons for children never having insurance seem amenable to interventions, including language barriers, hassles, better understanding of eligibility criteria, more responsive Medicaid/CHIP systems, and reducing premiums and copays.

The study findings indicate that uninsurance takes a major toll on children’s health, and that many have SHCNs. Approximately 40 % of children have suboptimal health, 2/3 have SHCNs, and four in five parents worry about their child’s health more than others. These findings suggest that insuring these uninsured children has the potential to improve children’s health while reducing parental worry.

Substantial access barriers and unmet needs were noted. Although RCTs are needed, evidence from observational studies indicates insuring uninsured children improves access, reduces unmet needs, and increases preventive and acute visits [14–16, 18, 41–43]. This is the first study, however, to our knowledge, to use primary data from in-depth surveys to identify considerable unmet needs for uninsured children with SHCNs, including unmet needs for 100 % of children requiring hearing aids/hearing care, and most children needing physical/occupational/speech therapy, mental healthcare, mobility aids, and home healthcare.

Identified racial/ethnic differences have implications for providing care for uninsured children. The findings suggest that for clinicians and health systems caring for uninsured Latino children, it may prove particularly useful to reduce parental worry about children’s health and out-of-pocket costs, and ensure that children have PCPs, regular sources of preventive care, and 24-h phone sick-care coverage. For clinicians and health systems caring for uninsured African-American children, it may prove particularly useful to focus on higher rates of asthma and ADHD, access to same-day sick visits without appointments, unmet acute-care and prescription needs, phone help/advice, reducing ED visits, improving providers’ understanding of parental preferences for raising children, and additional income needs for children’s medical expenses.

Certain study limitations should be noted. The research was conducted in urban settings in Texas, so the results may not necessarily generalize to non-urban settings or other states. Only Latinos and African-American children participated, so the findings may not generalize to uninsured children in other racial/ethnic groups. Outcomes were not assessed for insured or white children in the same communities, so comparisons between insured and uninsured children and whites and minorities could not be performed. Due to the length of the three surveys on insurance and healthcare, we were unable to ask questions regarding other public services accessed, such as WIC or public housing. The final sample size of 267 was relatively small, but is the largest community-based sample (to our knowledge) of uninsured children to be assessed for such a wide spectrum of outcomes.

Sizeable family financial burdens and costs of care were identified. Substantial proportions of parents reported needing additional income for children’s medical expenses, children’s illnesses causing family financial problems, and families reducing work hours to obtain children’s healthcare, and one in 10 parents ceased work because of the child’s health. This is particularly concerning, given that all of these children are in poor or low-income families with very limited financial means and reserves. This is further compounded by major out-of-pocket expenses for pediatric care, including a mean of $142/doctor visit, $47/preventive-care visit, $195/sick visit, $421/ED visit, and $593/hospitalization. Factoring in additional economic losses incurred by caring for sick children, including a mean wage loss of $343/day and child-care expenses of $156/day, it is clear that uninsured children are costly for parents, families, businesses, and local economies.

## Conclusions

Half of parents of uninsured minority children are unaware that their children are Medicaid/CHIP-eligible. These uninsured children have suboptimal health, impaired access to care, and major unmet needs for healthcare, mental healthcare, mobility aids/devices, dental care, specialty care, and vision care. The child’s health causes considerable family financial burden, and one in 10 parents ceased work because of their children’s poor health. These study finding indicate urgent needs for better parental education about Medicaid/CHIP and for improved Medicaid/CHIP outreach and enrollment. The data also indicate that special efforts should be made to target populations at highest risk of parental unawareness of children’s Medicaid/CHIP eligibility, including those uninsured the longest, those at the higher end of income eligibility, and Latinos.

For clinicians and health systems, the study results revealed racial/ethnic group-specific barriers which would seem to be crucial to target in efforts to improve the health and healthcare of uninsured minority children. For uninsured Latino children, these critical identified barriers include parental worry about children’s health and out-of-pocket costs, and children lacking PCPs, regular sources of preventive care, and 24-hour phone sick-care coverage. For uninsured African-American children, key identified barriers include higher rates of asthma and ADHD, lack of access to same-day sick visits without appointments, unmet acute-care and prescription needs, inadequate access to phone help/advice, high ED visit rates, limited healthcare provider understanding of parental preferences for raising children, and additional income needs for children’s medical expenses.

## Abbreviations

ADHD: attention-deficit hyperactivity disorder
CHIP: Children’s Health Insurance Program
ED: emergency department
LEP: limited English proficiency
PCP: primary-care provider
PedsQL: Pediatric Quality of Life Inventory Version 4.0 Generic Core Scales
RCT: randomized, controlled trial
SHCN: special healthcare need
